# Massive Scrotal Edema: An Unusual Manifestation of Obstructive Sleep Apnea and Obesity-Hypoventilation Syndrome

**DOI:** 10.1155/2013/685716

**Published:** 2013-02-21

**Authors:** Stephanie E. Dreifuss, Ernest K. Manders

**Affiliations:** ^1^School of Medicine, University of Pittsburgh, S532 Scaife Hall, 3550 Terrace Street, Pittsburgh, PA 15213, USA; ^2^Department of Plastic and Reconstructive Surgery, University of Pittsburgh Medical Center, Pittsburgh, PA, USA

## Abstract

Obstructive sleep apnea (OSA) may occur in association with obesity-hypoventilation (Pickwickian) syndrome, a disorder of ventilatory control affecting individuals with morbid obesity. Through the pressor effects of chronic hypercapnia and hypoxemia, this syndrome may result in pulmonary hypertension, right heart failure, and massive peripheral edema. We present a case of severe scrotal edema in a 36-year-old male with OSA and obesity-hypoventilation syndrome. A tracheostomy was performed to relieve hypoxemia and led to dramatic improvement of scrotal edema. No scrotal surgery was necessary. Followup at two months showed complete resolution of scrotal edema, improvement in mental status, and normalization of arterial blood gas measurements. This case demonstrates that OSA and obesity-hypoventilation syndrome may present with massive scrotal edema. Furthermore, if OSA is recognized as the cause of right heart failure, and if the apnea is corrected, the resultant improvement in cardiac function may allow reversal of massive peripheral, including scrotal, edema.

## 1. Background

Obstructive sleep apnea (OSA) is a syndrome characterized most often by symptoms of loud snoring, breathing interruptions or awakenings during sleep, and excessive daytime sleepiness [1]. In addition, patients with OSA may complain of morning headaches, decreased libido, intellectual deterioration or memory deficits, and depression [2]. Unusual signs and symptoms associated with OSA include delirium, enuresis, dysarthria, and seizure disorder [3, 4]. Patients with OSA demonstrate repetitive pharyngeal occlusion during sleep, resulting in apnea (breath cessation for at least ten seconds) or hypopnea (decreased ventilation). Consequences of these types of sleep-disordered breathing include nocturnal hypoxemia, cardiac arrhythmias, and sleep fragmentation leading to daytime hypersomnolence.

Most cases of OSA are idiopathic, but certain predisposing conditions have been recognized. Some of these disorders include obesity, macroglossia, nasal obstruction, adenoid and tonsillar hypertrophy, retrognathia, micrognathia, acromegaly, and hypothyroidism. Other risk factors include congestive heart failure, pulmonary hypertension, type II diabetes, and treatment refractory hypertension [1, 5]. Many different medical and surgical treatment modalities have been proposed to treat OSA, ranging from simple weight loss programs to reconstructive and diverting surgical procedures of the airway. Each of these methods has been met with varying degrees of success. Nasal continuous positive airway pressure (nasal CPAP) is usually effective and is now the treatment of choice in all but severe cases [1, 6].

Obesity-hypoventilation syndrome (OHS), sometimes called Pickwickian syndrome, is a disorder of ventilatory control that occurs in a subset of individuals with morbid obesity. It is characterized by chronic hypercapnia and hypoxemia as a result of blunted ventilator drive and the impediment to breathing imposed by obesity. Erythrocytosis, cyanosis, and cor pulmonale are commonly observed. Most patients with OHS also suffer from OSA. This combination of disorders may lead to profound hypoxemia with resultant pulmonary hypertension and right heart failure secondary to the pressor effects of hypoxemia and acidemia. In some cases, pulmonary hypertension may also result from left heart failure, a common finding in severe obesity [7].

## 2. Case Presentation

B.P. was a 36-year-old man at the time of presentation to the plastic surgery service for management of massive scrotal edema of three years' duration. This morbidly obese gentleman described a chronic course of slowly enlarging scrotum, abdomen, and lower extremities. His scrotal size made walking difficult and required specially tailored trousers. During this period, he had been hospitalized three times with diagnoses including cardiomegaly, right ventricular strain, polycythemia, obstructive sleep apnea, possible chronic obstructive pulmonary disease, lower extremity cellulitis, and elephantiasis.

On admission, the patient demonstrated a dusky color, mental dullness, and a tendency to fall asleep, but denied shortness of breath, chest pain, orthopnea, paroxysmal nocturnal dyspnea, or palpitations. In response to questions regarding his sleep, he related that he had been told he snored very loudly, but had no information about nocturnal breath cessation. He alleged that he slept four to twelve hours a night and denied any morning somnolence or headache. 

**Figure 1:**
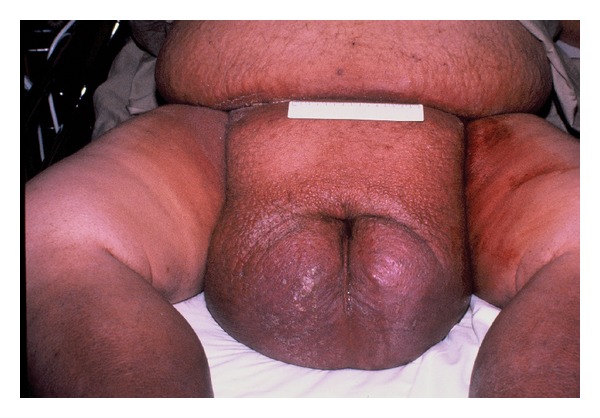
Our patient at admission. When standing, the scrotum descended to his knees and interfered with ambulation.

On physical examination his blood pressure was 130/90, his pulse was 95, his respiratory rate was 24, and he was afebrile. His weight was 198 kg, and his height was 178 cm. He demonstrated severe obesity, and intertrigo was evident in the large lower abdominal folds and in the groin. Direct examination of the oropharynx showed a large tongue, tonsils, uvula, and soft palate. Direct examination and nasal endoscopic examination revealed significantly reduced caliber of the oropharynx. There were no external or intrinsic masses, exudates, or ulcers. The neck was extremely thickened. Auscultation of the lungs revealed end-expiratory wheezing throughout the lung fields. There were no crackles. He showed severe edema of his lower legs, and a scrotum of massive proportions (Figure 1). The skin over the lower abdomen, scrotum, and lower extremities showed a peau d'orange appearance. The penis was not visible, as the enlarged scrotum extended so far forward that there was only a slit through which urine issued during voiding. The scrotum measured approximately 30 cm by 60 cm in dimension. The testes were not palpable. Deep pitting edema was present from the upper thighs downward, and chronic venous stasis changes of the lower extremity were noted.

Laboratory data included a hemoglobin of 19.5 g/dL with a hematocrit of 59.8%. Arterial blood gasses on room air were pH 7.31, pCO_2_ 63.2 mm Hg, pO_2_ 34 mm Hg, and bicarbonate 31.9 mEq/L. The hemoglobin was 53% saturated, and the alveolar-arterial oxygen tension gradient was 50 mm Hg. Pulmonary function testing revealed a mixed pattern of dysfunction with severe restriction and mild obstruction. The forced vital capacity (FVC) was 2.10 L (37% of predicted value), the forced expiratory volume in one second (FEV_1_) 1.44 L (34% of predicted value), and the FEV_1_/FVC ratio was 68%.

Pulmonary medicine and cardiology consultations were immediately sought. The patient was placed on continuous monitoring with ECG and pulse oximetry monitors. Chest X-ray revealed diffuse bilateral alveolar infiltrates and an enlarged heart. ECG demonstrated sinus rhythm of 100 beats per minute, right ventricular hypertrophy, and a right bundle branch block. Medical therapy with diuretics and inotropic agents was begun. Supplemental oxygen was initially withheld because even very low flow rates resulted in marked progression of his hypercapnia and respiratory acidemia.

During the first few days of his hospitalization, our patient demonstrated daytime hypersomnolence, confusion, and extreme irritability—mental status changes attributed to his prolonged hypoxia. During sleep at night, he was noted to snore loudly and experience mild chest collapse on inspiration. Apneic episodes of variable duration were noted at the bedside with the patient demonstrating inspiratory effort and a lack of air movement. After several days, nocturnal oxygen was administered by a 24% Venturi oxygen mask. Despite this therapy, fingertip pulse oximetry revealed saturations in the 49–72% range.

Following this dramatic desaturation, preliminary diagnostic polysomnography was conducted during a daytime nap in the sleep laboratory and revealed severe obstructive sleep apnea with an average of 120 abnormal obstructed breathing events (apneas plus hypopneas) per hour of sleep, each lasting greater than 10 seconds. These events were associated with profound oxyhemoglobin desaturations to as low as 50% and frequent arousals and awakenings from sleep. Though this study allows for prompt diagnosis of OSA with high sensitivity, specificity, and accuracy, it does not allow for the observation of sufficient REM sleep to measure disordered breathing episodes during this stage [8]. However, in this patient's case, enough apneic episodes were observed to make the diagnosis of OSA based on this preliminary testing. Nighttime polysomnography was later conducted and confirmed that the patient was suffering from OSA. 

Tracheostomy was performed. Serial arterial blood gases subsequently demonstrated improved oxygenation. The patient was maintained on 24% humidified O_2_ at night by tracheostomy collar, and he began to make dramatic clinical improvement.

**Figure 2:**
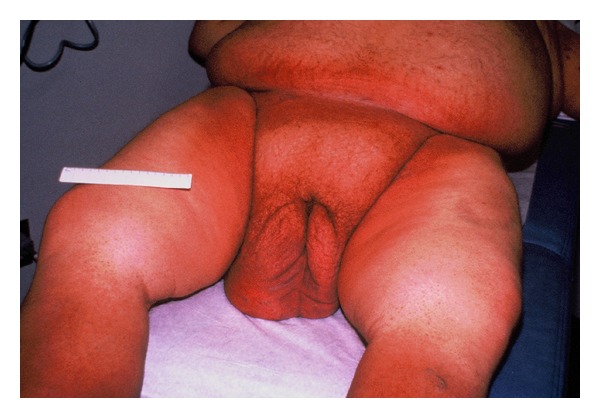
The appearance of the scrotum after tracheostomy and diuresis. The patient was able to ambulate.

Over the next two weeks in the hospital, this gentleman lost approximately 70 lbs. (31.8 kg). His mental status and mood improved, his exercise tolerance increased, and his lymphedematous scrotum decreased markedly in size (Figure 2). He was discharged on furosemide 40 mg PO daily, potassium supplementation, and nystatin powder for his intertrigo. The patient was able to walk comfortably and resume wearing trousers without modification.

Followup at two months demonstrated marked improvement in mental status. Arterial blood gases revealed normal pH and pCO_2_ and only mild hypoxemia (pO_2_ 66 mm Hg). Subsequent outpatient follow-up visits documented additional improvement and continued weight loss.

## 3. Discussion

In this paper, we present a case of massive scrotal edema in which the diagnosis went unrecognized for an extended period of time due to the unusual nature in which it presented. This patient demonstrated severe scrotal edema as a manifestation of right heart failure resulting from OSA and OHS.

Several of the unusual presentations of OSA have been successfully treated with nasal CPAP [1]. However, tracheostomy has been shown to be a viable alternative in treating morbidly obese patients with combined OSA and OHS [1, 6, 9]. In fact, Guilleminault et al. defined five criteria for tracheostomy in patients with OSA: (1) disabling sleepiness with severe socioeconomic impact; (2) severe cardiac arrhythmias with sleep apnea; (3) high apnea index (>60); (4) oxygen saturation less than 40% during sleep; and (5) no improvement in clinical symptoms and sequelae after medical trials [10]. According to Won et al., tracheostomy is considered a long-term, curative treatment of OSA in morbidly obese patients with obesity hypoventilation syndrome [11]. Indeed, the success of tracheostomy in treatment of OSA was clearly demonstrated in the case presented here.

The massive scrotal edema demonstrated by our patient is frequently encountered by the urologic surgeon. This condition has been attributed to several different etiologies, including medical causes (cardiac failure, renal disease, blood dyscrasias, and ascites), obstructive conditions (abdominal neoplasms, inguinal hernias, and thrombosis of spermatic vein), infectious diseases (filarial elephantiasis), iatrogenic interventions (radiation therapy, node dissection), and trauma [12, 13]. Important diagnostic clues for determining the etiology of scrotal edema include age of patient, acuteness of presentation, and presence of pain [14]. 

Because response to medical therapy is poor, surgical intervention is considered the best option for treatment of scrotal edema. Of the multiple operative procedures detailed, excisional therapy continues to remain the most prominent [15]. This technique, originally attributed to Delpech, involves dissection at the level of Buck's fascia on the penis and complete separation of the testicles and spermatic cord from the scrotum. This method is both expeditious and has enjoyed successful, reproducible, long-term results [15, 16]. Secondary coverage following dissection can be obtained from retained dorsal scrotal skin, split thickness skin grafting, or fasciocutaneous flaps from the thigh [15].

The case presented here is notable because it delivers two lessons. First, obstructive sleep apnea and obesity-hypoventilation syndrome may present with massive scrotal edema. This striking physical finding is the consequence of right heart failure thought to be due to pulmonary arterial hypoxemia and hypercapnia, which causes pulmonary arterial acidemia and vasoconstriction. The pulmonary hypertension resulting from these chemical stimuli leads to right heart failure [4]. Second, if obstructive sleep apnea and/or obesity-hypoventilation syndrome is recognized as the cause of right heart failure, and if the apnea is corrected, the resultant improvement in cardiac function may allow reversal of massive peripheral edema, including scrotal edema. Astute physicians and surgeons should recognize that scrotal edema occurring in patients with severe obstructive sleep apnea will likely be reversed upon the successful treatment of the sleep apnea. Indeed, no operative intervention should be entertained, at least initially.

We suggest that massive scrotal edema without obvious cause should prompt a physician or surgeon to ask whether the patient might be suffering from undiagnosed and untreated sleep apnea and obesity-hypoventilation syndrome.

## 4. Conclusion

Massive scrotal edema may result from right heart failure, as a consequence of severe obstructive sleep apnea and obesity-hypoventilation syndrome. In the patient presented here, tracheostomy to correct underlying obstructive sleep apnea produced improved oxygenation status, improved cardiac function, and a dramatic subsequent decrease in scrotal size. Corrective scrotal surgery did not prove necessary. Otherwise unexplained massive scrotal edema in a morbidly obese patient might prompt suspicion of the presence of severe obstructive sleep apnea with right heart failure.
